# Early tracheostomy in severe traumatic brain injury: an umbrella systematic review

**DOI:** 10.1016/j.bjane.2026.844727

**Published:** 2026-02-12

**Authors:** Raul Ribeiro de Andrade, Edla Vitória Santos Pereira, Igor Hudson Albuquerque e Aguiar, Olavo Barbosa de Oliveira Neto, João Gustavo Rocha Peixoto dos Santos, Fabiano Timbó Barbosa, Célio Fernando de Sousa-Rodrigues

**Affiliations:** a Universidade Federal de Alagoas (UFAL), Instituto de Ciências Biológicas e da Saúde, Maceió, AL, Brazil; b Universidade Federal de Alagoas (UFAL), Maceió, AL, Brazil; c Centro Universitário Cesmac, Faculdade de Medicina, Maceió, AL, Brazil; d Researcher - Center for Research in Applied Morphology (CIMA) - La Frontera University - Temuco, Chile; e Universidade de São Paulo (USP), São Paulo, SP, Brazil; f Universidade Federal de Alagoas (UFAL), Faculdade de Medicina, Maceió, AL, Brazil

**Keywords:** Epidemiology, Length of Stay, Mortality, Tracheostomy, Traumatic brain injury

## Abstract

**Background:**

Tracheostomy is an option to ensure airway safety in patients with severe traumatic brain injury. However, the optimal timing for tracheostomy remains unclear based on current evidence.

**Methods:**

Umbrella systematic review to determine the effectiveness of early tracheostomy in TBI. Databases: PubMed, Embase, Scopus, Web of Science, Lilacs, Cochrane, Open Grey, and clinical trials. Inclusion criteria: Meta-analysis of early tracheostomy in severe TBI patients. Exclusion criteria: if there was no data regarding the time of death or the follow-up period. Data extraction: Selection, risk of bias evaluation, and data extraction were performed by two independent authors.

**Results:**

Four meta-analyses were included from 5673 initial records, and a new meta-analysis was performed from data obtained in primary studies. The evidence included in this umbrella review showed that early tracheostomy reduced ICU (MD = -5.69 days; 95% CI [-7.78, -3.59]) and Hospital (MD = -3.53 days; 95% CI [-4.44, -2.62]) length of stay, time in mechanical ventilation (MD = -5.08; 95% CI [-7.12, -3.05]) and risk of ventilator associated pneumonia (RR = 0.78; 95% CI [0.70, 0.86],). These studies cannot determine the effectiveness of early tracheostomy on mortality (RR = 1.32; 95% CI [0.89, 1.96],) or neurological prognosis.

**Conclusions:**

This umbrella review suggests that early tracheostomy is effective in reducing ICU and Hospital length of stay, time in mechanical ventilation, and ventilator-associated pneumonia.

**Inplasy protocol:**

202280096.

## Introduction

Traumatic Brain Injury (TBI) is any injury that affects the skull, brain tissue, and its associated vessels, and it’s a major public health issue worldwide. The overall incidence of TBI is estimated at 1299 cases in North America, 1012 cases in Europe, and 801 cases in Africa (per 100,000 people).^1^ In Brazil, there were one million hospitalizations of TBI patients between 2010 and 2019, and 45,42% of those were in patients aged 20 to 49 years old.^2^

TBI can be classified by the Glasgow Coma Scale (GCS) as mild (13‒15), moderate (9‒12), and severe (< 9) (TEASDALE, 1974).^3^ One of the aims in assisting patients with severe TBI is the hemodynamic and airway management to avoid secondary injuries as hypoxemia and hypercapnia.^4^^,^^5^

Extubating in neurological patients remains a challenge. Waiting for full neurological recovery is not mandatory. However, the ability to cough, swallow, and maintain eye contact during evaluation can be assessed.^6^ This strategy can result in prolonged time in mechanical ventilation, and it has increased hospital morbidity and mortality.^7^^,^^8^ The prolonged hospital care is associated with complications, such as pneumonia, thromboembolic events, and mortality.^9^

In this scenario, tracheostomy is one of the options to guarantee a safer airway, promote early patient mobilization, progression of the diet, reduction of airway resistance, and complications ratio.^10^ Although the time to perform tracheostomy still remains unclear in the light of current evidence.^6^^,^^11^

Hence, the aim of this umbrella review was to determine the effectiveness of early tracheostomy in severe patients with traumatic brain injury.

## Methods

The Preferred Reporting Items for Systematic Review and Meta-analysis Protocols^12^ was used to design a protocol, which was registered on Inplasy – International Platform of Registered Systematic Review and Meta-analysis Protocols (ID number: 202280096).^13^

The inclusion criteria were: (P) Patients above 18 years old with a severe traumatic brain injury and advanced airway support; (I) Early tracheostomy (< 10 days of intubation); (C) Late tracheostomy (> 10 days) or prolonged intubation; (O) Mortality, time on ICU stay, on Hospital stay and in mechanical ventilation, complications (pneumonia, pressure ulcers, thromboembolic events and time using antibiotics), and quality of life (scores regarding neurological functions); and (S) Systematic reviews with meta-analysis. No language restrictions were applied. A study would be excluded if there was no data regarding the time of death and follow-up period in hospital stay or after discharge.

Online databases were searched on August 22^nd^, 2022, using the MESH terms of Craniocerebral Trauma and tracheostomy: Medline by PubMed, Lilacs, Cochrane, Scopus by Elsevier, Web of Science, and Embase by Elsevier. The references of the selected studies were also analyzed. Grey literature was sought with SIGLE by Open Grey and Clinical Trial Register at the International Clinical Trials Registry Platform (Supplementary Material).

Two independent reviewers (RRA and IHAA) selected the studies and performed data extraction using pre-established forms.^13^ Then, disagreements were solved by consensus meetings with a third and more experienced reviewer (OBON).

Risk of bias was assessed using the ROBIS tool. This evaluation was performed by two reviewers, independently (RRA and EVSP). Cohen’s kappa statistic was used to measure the level of agreement between reviewers for the selection of eligible studies and for the risk of bias assessment. MetaXL 5.3 (Epigear, Queensland, Australia) was used to perform meta-analyses. The Relative Risk (RR) was calculated for dichotomic outcomes and the Mean Difference (MD) for continuous outcomes (Confidence Interval 95%). Predicting a possible heterogeneity between studies, the random effects model was used.

To avoid the results being inflated by overlap of primary studies in the included meta-analyses, we performed our own meta-analyses with the primary studies' data (Supplementary Material). The presence of heterogeneity was analyzed by the Cochrane Q statistic and was measured using the Higgins Test (I^2^). To explore heterogeneity, we performed a sensitivity analysis by excluding studies with a high risk of bias, and a subgroup analysis by comparing prospective versus retrospective cohorts and late tracheostomy versus prolonged intubation in the control group. The publication bias was assessed with the DOI-plot and LFK index. We also checked and didn’t find any retractions in the selected studies.

GRADE approach (Grading of Recommendations Assessment, Development and Evaluation) was used to assessing certainty of the evidence.

## Results

**Figure 1:**
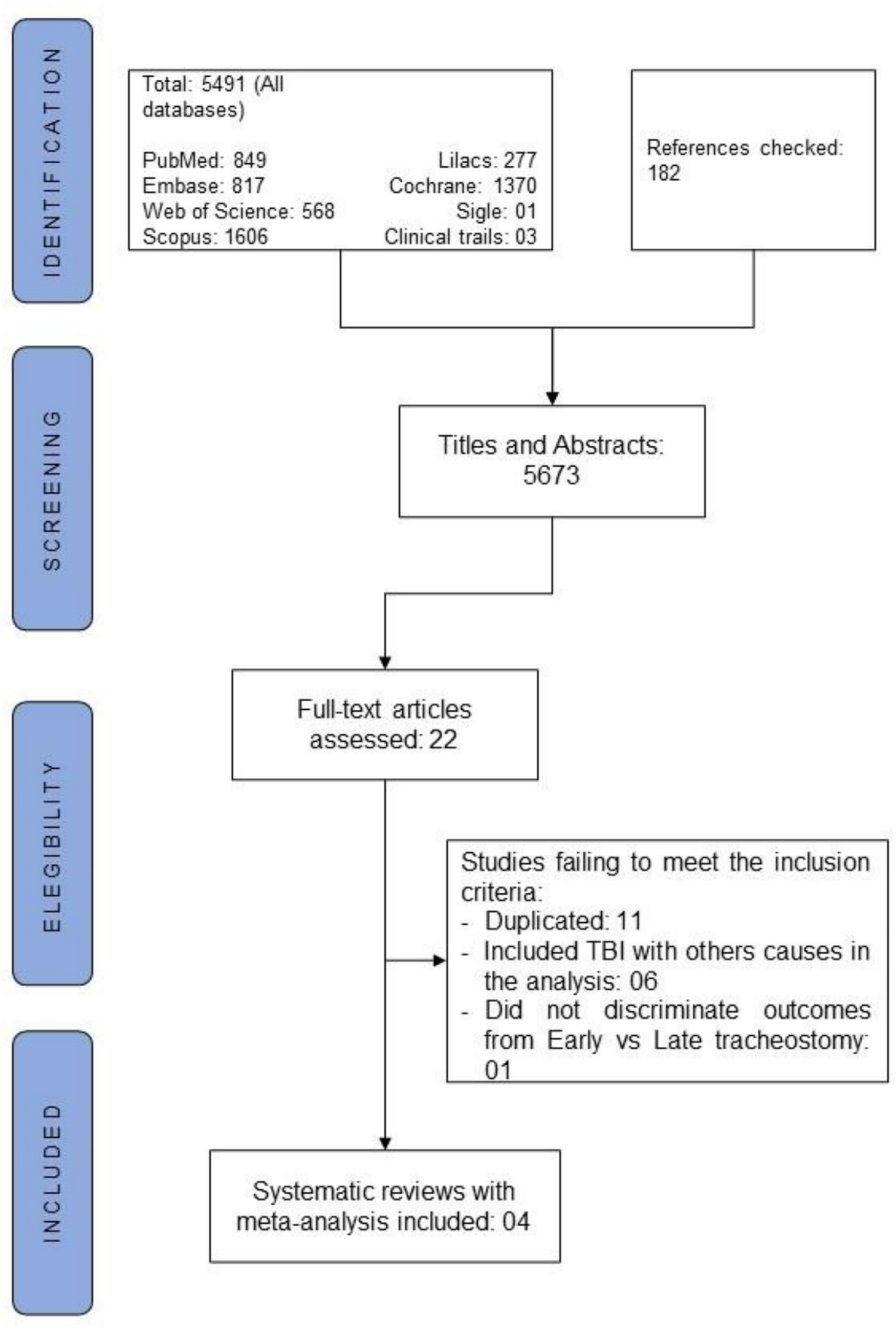
Flowchart of the selection process.

In total, 5491 registers were identified from the search strategy across all online databases. Then, 22 articles were identified as potentially relevant to this umbrella review. In the selection process, 18 articles failed to meet the inclusion criteria, as 11 articles were duplicated, six papers included TBI with other causes of mechanical ventilation in the analysis, and one publication did not discriminate outcomes from early versus late tracheostomy. We did not identify new studies in the screening process of references (Fig. 1). Thus, four articles^14^, ^15^, ^16^, ^17^ were included in this umbrella review (agreement: 91.3%; Kappa = 0.774, 95% CI 0.41‒1.00) (Table 1).

**Table 1:** Results of meta-analyses included with primary studies.

Outcome	Meta-analysis	Selected primary studies	Results
Mortality	McCredie et al. (2017)^14^	**RCT** Sugerman et al. (1997)^18^ Bouderka et al. (2004)^19^ Dunhan et al. (2014)^20^ Barquist et al. 2006^32^	RCT: RR = 1.2 [0.44, 3.30], I^2^ = 37%
	Lu et al. (2018)^15^	**RCT** Sugerman et al. (1997)^18^ Bouderka et al. (2004)^19^ Dunhan et al. (2014)^20^	RCT: OR = 2.58 [0.96, 6.96], I^2^ = 0%
		**Observational** Siddiqui et al. (2015)^22^ Kahlili et al. (2017)^24^ Alali et al. (2014)^26^ Ahmed, Kuo et al. (2007)^27^ Wang et al. (2012)^28^	Cohort: OR = 1.15 [0.81, 1.63], I^2^ = 0%
	Franca et al. (2020)^16^	**RCT** Dunhan et al. (2014)^20^	RCT and Cohort: Risk = 0.03 [-0.02, 0.07], I^2^ = 69%
		**Observational** Shibahashi et al. (2017)^21^ Kahlili et al. (2017)^24^ Alali et al. (2014)^26^ Ahmed, Kuo et al. (2007)^27^	
	Marra et al. (2021)^17^	**RCT** Sugerman et al. (1997)^18^ Dunhan et al. (2014)^19^	RCT: OR = 3.154 [0.456, 21.695], I^2^ = 0%
		**Observational** Shibahashi et al. (2017)^21^ Robba et al. (2020)^23^ Kahlili et al. (2017)^24^ Rizk et al. (2011)^25^ Alali et al. (2014)^26^ Ahmed, Kuo et al. (2007)^27^	Cohort: OR 1.505 [0.993, 2.279], I^2^: 49.435%
VAP	McCredie et al. (2017)^14^	**RCT** Sugerman et al. (1997)^18^ Bouderka et al. (2004)^19^ Dunhan et al. (2014)^20^ Barquist et al. (2006)^32^ Blot et al. (2008) Fayed et al. (2012)	RR = 0.89 [0.65, 1.21], I^2^ = 54%
	Lu et al. (2018)^15^	**RCT** Sugerman et al. (1997)^18^ Bouderka et al. (2004)^19^ Dunhan et al. (2014)^20^	RCT: OR = 0.89 [0.47, 1.68], I^2^ = 0%
		**Observational** Siddiqui et al. (2015)^22^ Kahlili et al. (2017)^24^ Alali et al. (2014)^26^ Ahmed, Kuo et al. (2007)^27^ Wang et al. (2012)^28^	Cohort: OR = 0.62 [0.51, 0.77], I^2^ = 0%
	Franca et al. (2020)^16^	**RCT** Dunhan et al. (2014)^20^	RCT and Cohort: RR = 0.78 [0.70, 0.88], I^2^ = 0%
		**Observational** Shibahashi et al. (2017)^21^ Kahlili et al. (2017)^24^ Alali et al. (2014)^26^ Ahmed, Kuo et al. (2007)^27^	
	Marra et al. (2021)^17^	**Observational** Shibahashi et al. (2017)^21^ Robba et al. (2020)^23^ Kahlili et al. (2017)^24^ Alali et al. (2014)^26^ Ahmed, Kuo et al. (2007)^27^ Wang et al. (2012)^28^	Cohort: OR = 0.623 [0.518, 0.750], I^2^ = 0%
Time in MV	McCredie et al. (2017)^14^	**RCT** Sugerman et al. (1997)^18^ Bouderka et al. (2004)^19^ Dunhan et al. (2014)^20^ Barquist et al. (2006)^32^ Blot et al. (2008) (NO TBI) Terragni et al. (2010) (NO TBI) Fayed et al. (2012) (NO TBI) Bösel et al. (2013) (NO TBI) Youngi et al. (2021) (NO TBI)	MD = -2.72 [-4.15, -2.19], I^2^ = 0%
	Lu et al. (2018)^15^	**RCT** Bouderka et al. (2004)^19^ Dunhan et al. (2014)^20^	RCT and Cohort: MD = -4.92 [-6.82, -3.02], I^2^ = 51%
		**Observational** Siddiqui et al. (2015)^22^ Robba et al. (2020)^23^ Kahlili et al. (2017)^24^ Alali et al. (2014)^26^ Ahmed, Kuo et al. (2007)^27^ Wang et al. (2012)^28^	
	Franca et al. (2020)^16^	**RCT** Dunhan et al. (2014)^20^	RCT and Cohort: MD = -4.15 [-6.30, -1.99], I^2^ = 85%
		**Observational** Shibahashi et al. (2017)^21^ Alali et al. (2014)^26^ Ahmed, Kuo et al. (2007)^27^	
	Marra et al. (2021)^17^	**RCT** Dunhan et al. (2014)^20^	RCT and Cohort: MD = -4.866 [-6.981, -2.751], I^2^ = 93.203%
		**Observational** Shibahashi et al. (2017)^21^ Robba et al. (2020)^23^ Alali et al. (2014)^26^ Ahmed, Kuo et al. (2007)^27^	
LOS (ICU)	McCredie et al. (2017)^14^	**RCT** Sugerman et al. (1997)^18^ Blot et al. (2008) (NO TBI) Terragni et al. (2010) (NO TBI) Bösel et al. (2013) (NO TBI) Youngi et al. (2021) (NO TBI)	MD = -2.55 [-4.59, -0.50], I^2^ = 0%
	Lu et al. (2018)^15^	**RCT** Sugerman et al. (1997)^18^ Bouderka et al. (2004)^19^ Dunhan et al. (2014)^20^	RCT and Cohorts: MD = -3.08 [-3.75, -2.41], I^2^ = 38%
		**Cohorts** Siddiqui et al. (2015)^22^ Kahlili et al. (2017)^24^ Ahmed, Kuo et al. (2007)^27^ Wang et al. (2012)^28^	
	Franca et al. (2020)^16^	**Cohorts** Shibahashi et al. (2017)^21^ Kahlili et al. (2017)^24^ Alali et al. (2014)^26^ Ahmed, Kuo et al. (2007)^27^	Cohorts: MD = -5.87 [-8.74, -3.00], I^2^ = 83%
	Marra et al. (2021)^17^	**Cohorts** Shibahashi et al. (2017)^21^ Robba et al. (2020)^23^ Kahlili et al. (2017)^24^ Alali et al. (2014)^26^ Ahmed, Kuo et al. (2007)^27^	Cohorts: MD = -5.96 [-7.99, -3.92], I^2^ = 88.661%
LOS (Hospital)	Lu et al. (2018)^15^	**Cohorts** Siddiqui et al. (2015)^22^ Kahlili et al. (2017)^24^ Alali et al. (2014)^26^ Ahmed, Kuo et al. (2007)^27^ Wang et al. (2012)^28^	Cohorts: MD = -4.79 [-8.63, -0.94]; I^2^ = 59%
	Franca et al. (2020)^16^	**Cohorts** Shibahashi et al. (2017)^21^ Kahlili et al. (2017)^24^ Alali et al. (2014)^26^	Cohorts: MD = -6.68 [-8.03, -5.32]; I^2^ = 0%
	Marra et al. (2021)^17^	**Cohorts** Shibahashi et al. (2017)^21^ Robba et al. (2020)^23^ Kahlili et al. (2017)^24^ Alali et al. (2014)^26^ Ahmed, Kuo et al. (2007)^27^	Cohorts: MD = -6.97 [-8.25, -5.68]; I^2^ = 0%

RR, Risk Ratio; OR, Odds Ratio; MD, Mean Difference; RCT, Randomized Control Trial; VAP, Ventilator-Associated Pneumonia; Time in MV, Duration (in days) of mechanical ventilation; LOS (ICU), ICU Lenght of stay; LOS (Hospital), Hospital Lenght of stay; ET, Early Tracheostomy; LT, Late Tracheostomy; PI, Prolonged Intubation.

### Risk of bias

There were 70% of agreement on this stage (Kappa = 0.400, 95% CI 0.166‒0.684). Disagreements were solved by a third reviewer (OBON):

Domain 1: Only McCredie et al. (2017)^14^ published a priori protocol, nonetheless Lu et al. (2018),^15^ Franca et al. (2020)^16^ and Marra et al. (2021)^17^ had describe satisfactory the eligibility criteria and outcomes; Domain 2: Franca et al. (2020)^16^ and Marra et al. (2021)^17^ presented high concerns regarding the selection process because was restrict to one database (PubMed) and the search strategy was not clear enough to replicate; Domain 3: all of the included studies^14^, ^15^, ^16^, ^17^ had low risk of bias because data extraction and risk of bias was performed by two reviewers independently; Domain 4: Franca et al. (2020)^16^ and Marra et al. (2021)^17^ didn’t take the high heterogeneity in consideration in their results and conclusions, Lu et al. (2018)^15^ wasn’t clear about the synthesis and just McCredie et al. (2017)^14^ had low concerns for executed what was planned in protocol.

### Mortality

The meta-analyses results by McCredie et al. (2017),^14^ Lu et al. (2018),^15^ Franca et al. (2020)^16^ and Marra et al. (2021)^17^ reported no difference in mortality between early and late tracheostomy groups (Table 1). In our meta-analysis we also found no difference between groups with three RCTs^18^, ^19^, ^20^ and this data showed no heterogeneity (RR = 1.86 [0.90, 3.84], I^2^ = 0%, *Q* = 1.06, p = 0.59). The same result was presented with eight cohorts,^20^, ^21^, ^22^, ^23^, ^24^, ^25^, ^26^, ^27^ but this data reported a moderate heterogeneity (RR = 1.32 [0.89, 1.96], I^2^ = 56%, *Q* = 16.06, p = 0.02) (Table 2).

**Table 2:** GRADE.

Outcome	Study	ET group	LT/PI group	Day of ET	N° of participants (studies)	Certainty of the evidence (GRADE)	Meta-analysis
Mortality	**RCT**						
	Bouderka et al. (2004)^19^	12/31	7/31	5‒6 days	153 (3 RCTs)	⨁⨁◯◯ Low^a^	RR = 1.86 [0.90, 3.84], I^2^ = 0%
	Dunhan et al. (2014)^20^	0/15	0/9	3‒5 days			
	Surgerman et al. (1997)^18^	5/35	1/32	3‒5 days			
	**Cohorts**						
	Ahmed & Kuo et al. (2007)^27^	4/27	1/28	≤ 7 days	5043 (7 Cohorts)	⨁◯◯◯ Very Low^a^	RR = 1.32 [0.89, 1.96], I^2^ = 56%
	Alali et al. (2014)^26^	48/571	39/571	≤ 8 days			
	Shibahashi et al. (2017)^21^	1/40	4/51	≤ 72 hours			
	Rizk et al. (2011)^25^	238/1577	111/1527	≤ 7 days			
	Wang et al. (2012)^28^	2/16	4/50	≤ 7 days			
	Siddiqui et al. (2015)^22^	4/49	9/51	≤ 7 days			
	Khalili et al. (2017)^24^	10/53	18/99	≤ 6 days			
VAP	**RCT**						
	Bouderka et al. (2004)^19^	12/31	7/31	5‒6 days	213 (3 RCTs)	⨁⨁◯◯ Low^a^	RR = 0.94 [0.70, 1.27]; I^2^ = 0%
	Dunhan et al. (2014)^20^	0/15	0/9	3‒5 days			
	Surgerman et al. (1997)^18^	5/35	1/32	3‒5 days			
	**Cohorts**						
	Ahmed & Kuo et al. (2007)^27^	11/27	14/28	≤ 7 days	2039 (7 Cohorts)	⨁⨁◯◯ Low^b^	RR = 0.78 [0.70, 0.86]; I^2^ = 0%
	Alali et al. (2014)^26^	238/571	301/571	≤ 8 days			
	Shibahashi et al. (2017)^21^	13/40	21/51	≤ 72 hours			
	Wang et al. (2012)^28^	7/16	38/50	≤ 7 days			
	Siddiqui et al. (2015)^22^	22/49	32/51	≤ 7 days			
	Khalili et al. (2017)^24^	28/53	59/99	≤ 6 days			
	Robba et al. (2020)^23^	49/180	100/253	≤ 7 days			
Time in MV	**RCT**						
	Bouderka et al. (2004)^19^	14.5 ± 7.3d / 31p	17.5 ± 10.6d / 31p	5‒6 days	86 (2 RCTs)	⨁⨁◯◯ Low^a^	
	Dunhan et al. (2014)^20^	14.1 ± 5.7d / 15p	19 ± 11.3d / 32 p	3‒5 days			MD = -2.95 [-6.16, 0.26]; I^2^ = 0%
	**Retrospective cohorts**						
	Ahmed & Kuo et al. (2007)^27^	15.7 ± 6d / 27p	25.8 ± 11.8d / 28p	≤ 7 days	1288 (3 cohorts)	⨁⨁⨁◯ Moderate^c^	
	Alali et al. (2014)^26^	21.4 ± 10.45d/571p*	24.9 ± 5.95d/ 571p*	≤ 8 days			MD = -3.26 [-3.94, -2.57]; I^2^ = 0%
	Shibahashi et al. (2017)^21^	5 ± 1.54d / 40p*	8 ± 3.05d/ 51p*	≤ 72 hours			
	**Prospective cohorts**						
	Wang et al. (2012)^28^	13.7 ± 7.3d / 16p	23.4 ± 11d / 50p	≤ 7 days	499 (2 cohorts)	⨁⨁⨁⨁ High^d^	MD = -7.53 [-9.05, -6.01]; I^2^ = 0%
	Robba et al. (2020)^23^	12.35 ± 6.73d/180p*	19.63 ± 10.29d/253p*	≤ 7 days			
ICU lenght of stay	**RCT**						
	Surgerman et al. (1997)^18^	16 ± 5.9d / 35p	19 ± 11.3d / 32p	3‒5 days			
	**Retrospective cohorts**						
	Ahmed & Kuo et al. (2007)^27^	19 ± 7.7d / 27p	25.8 ± 11.8d / 28p	≤ 7 days			
	Alali et al. (2014)^26^	13.7 ± 5.95d / 571p*	19.7 ± 7.43 / 571p*	≤ 8 days	1288 (3 Cohorts)	⨁⨁⨁◯ Moderate^e^	MD = -4.67 [-7.85, -1.5]; I^2^ = 0%
	Shibahashi et al. (2017)^21^	10 ± 4.61d / 40p*	12.06 ± 3.81d / 51p*	≤ 72 hours			
	**Prospective cohorts**						
	Wang et al. (2012)^28^	14.9 ± 8.9d / 16p	22.1 ± 7.6d / 50p	≤ 7 days	651 (3 Cohorts)	⨁⨁⨁◯ Moderate^f^	MD = -7.34 [-9.76, -4.92]; I^2^ = 0%
	Khalili et al. (2017)^24^	26.79 ± 13.16d/ 53p	34.92 ± 20.07d/ 99p	≤ 6 days			
	Robba et al. (2020)^23^	19.6 ± 19.9d / 180p	26.7 ± 12.5d / 253p	≤ 7 days			
Hospital lenght of stay	**Cohorts**						
	Ahmed & Kuo et al. (2007)^27^	24.36 ± 5.48d / 27p*	28 ± 6,25d / 28p*	≤ 7 days	1939 (6 Cohorts)	⨁⨁⨁◯ Moderate^f^	MD = -3.53 [-4.44, -2.62]; I^2^ = 0%
	Alali et al. (2014)^26^	21.4 ± 10.41d/571p*	24.9 ± 5.95/571p*	≤ 8 days			
	Shibahashi et al. (2017)^21^	52.64 ± 19.22d/40p*	56.29 ± 16.78d/51p	≤ 72 hours			
	Wang et al. (2012)^28^	38.0 ± 21.4d/ 16p	46.8 ± 22d / 50p	≤ 7 days			
	Robba et al. (2020)^23^	35.1 ± 34.4d /180p	34.7 ± 33.6d / 253 p	≤ 7 days			
	Khalili et al. (2017)^24^	38.58 ± 20.18d/ 53p	46.40 ± 24.56d/ 99p	≤ 6 days			

a Small number of events, Large IC.

b LFK index: -2,10 (Major asymmetry), I^2^ = 0%.

c High magnitude of effect, I^2^ = 0%, LFK index: -2,71 (Major asymmetry).

d No asymmetry, I^2^ = 0%, high magnitude of effect.

e High magnitude of effect, High heterogeneity.

f High magnitude of effect, I^2^ = 0% (Major asymmetry). ET, Early Tracheostomy; LT, Late Tracheostomy; PI, Prolonged Intubation; p, Participants. Data in Mean ± Standard Deviation / participants.

⁎ Data converted of Median and Interquartile interval from primary studies by Wan et al. (2014).^39^

### Ventilator-associated pneumonia

**Figure 2:**
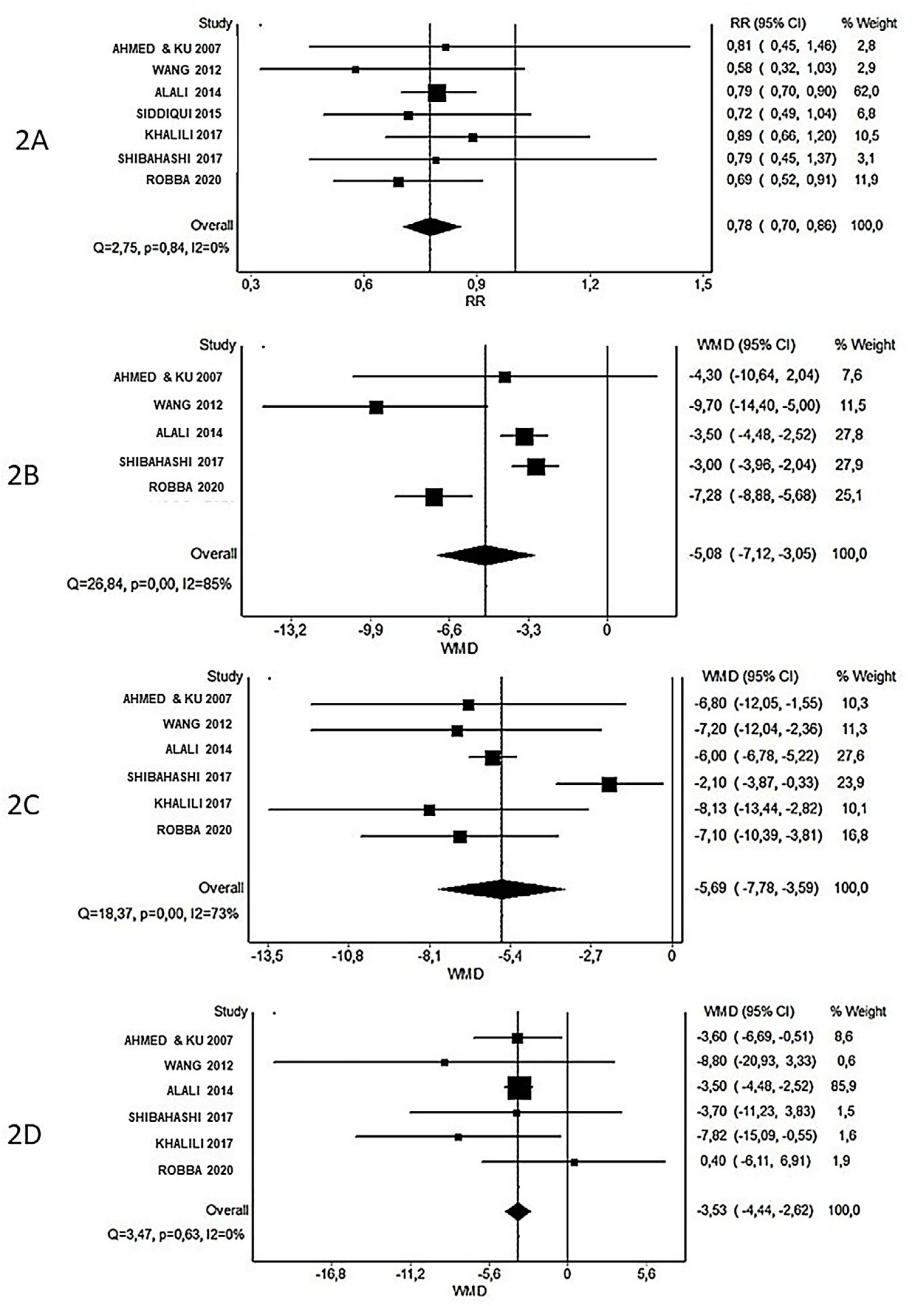
Forest plot of early tracheostomy in severe TBI patients: (A) Ventilator-associated pneumonia, (B) Time in mechanical ventilation, (C) ICU length of stay, (D) Hospital length of stay.

Franca et al. (2020)^16^ and Marra et al. (2021)^17^ reported a decrease in the risk of ventilator-associated pneumonia in the ET group. Otherwise, McCredie et al. (2017)^14^ and Lu et al. (2018)^15^ reported no difference between groups. Our meta-analyses found no difference between early and late tracheostomy on the risk of pneumonia (RR = 0.94 [0.70, 1.27]; I^2^ = 0%, *Q* = 0.07, p = 0.97) across three RCTs.^18^, ^19^, ^20^ The ET group reduced in 22% the risk of ventilator-associated pneumonia in seven cohort studies^21^, ^22^, ^23^, ^24^, ^25^, ^26^, ^27^ (RR = 0.78 [0.70, 0.86]; I^2^ = 0%, *Q* = 2.75, p = 0.84). Meta-analyses with RCTs and cohort studies did not show statistical heterogeneity (Fig. 2).

### Duration of mechanical ventilation

All included meta-analyses^14^, ^15^, ^16^, ^17^ reported that the ET group was significantly associated with reduced duration of mechanical ventilation. In our analysis, there was no difference in the Mean Difference in days of mechanical ventilation between groups in two RCTs^18^^,^^20^ (MD = -2.95 [-6.16, 0.26]; (I^2^ = 0%, *Q* = 0.00, p = 0.98). In our meta-analysis of five cohorts,^21^^,^^23^^,^^26^, ^27^, ^28^ the ET group was associated with fewer days in mechanical ventilation, although this result was based on high heterogeneity (MD = -5.08 [-7.12, -3.05]; I^2^ = 85%, *Q* = 26.84, p = 0.00) (Fig. 2).

### ICU length of stay

Four meta-analyses^14^, ^15^, ^16^, ^17^ reported that the ET group was significantly associated with reduced ICU length of stay. In our analysis, just one RCT^18^ evaluated this outcome. The ET group was associated with fewer days in mechanical ventilation in six cohorts^21^^,^^23^^,^^24^^,^^26^, ^27^, ^28^ (MD = -5.69 [-7.78, -3.59]; I^2^ = 73%, *Q* = 18.37, p = 0.00) (Fig. 2).

### Hospital length of stay

McCredie et al. (2017),^14^ Lu et al. (2018),^15^ and Franca et al. (2020)^16^ reported that the ET group was significantly associated with reduced hospital length of stay. In our meta-analysis, we also reported that the ET group was associated with fewer days in mechanical ventilation in six cohorts^21^^,^^23^^,^^24^^,^^26^, ^27^, ^28^ (MD = -3.53 [-4.44, -2.62]; I^2^ = 0%, *Q* = 3.47, p = 0.63) (Fig. 2).

### Quality of life

None of the included meta-analyses reported this outcome. However, five cohort studies presented some types of scores for this outcome. Two cohorts^21^^,^^22^ demonstrated by GOS (Glasgow Outcome Scale), Shibahashi et al. (2017)^21^ reported no statistical difference between groups, and Siddiqui et al. (2015)^22^ showed a better result in the early tracheostomy group, but without a statistical analysis.

Two cohorts^23^^,^^24^ evaluated GOSE (Glasgow Outcome Scale-Extended) at 6 months of follow-up. Robba et al. (2020)^23^ reported a worse result (OR = 1.69 [1.07–2.67], p = 0.018). Khalili et al. (2017)^24^ showed no statistical difference between groups. One study^25^ evaluated FIM (Functional Independence Measure) with better results in the ET group (FIM > 10, ET: 43% vs. LT: 29%, p < 0.0001).

### Other health care related outcomes

None of the meta-analyses selected reported these outcomes. One cohort^26^ reported fewer events of decubitus ulcer (ET: 4.03% vs. LT: 8.93%), deep venous thrombosis (ET: 8.23% vs. LT: 14.36%), and pulmonary embolism (ET: 1.75% vs LT: 3.33%) in the ET group. Another cohort^25^ showed more infectious events (sepsis, septicemia, and acute sinusitis) in the LT group (ET: 24.35% vs. LT: 34.9%). Moreover, Robba et al. (2020)^23^ presented a higher need for antibiotics in the LT group (ET: 88.33% vs. LT: 95.65%).

### Publication bias

The risk of publication bias was analyzed in mortality and VAP from RCTs.^18^, ^19^, ^20^ In the mortality meta-analysis, we found no asymmetry in publication bias (LFK index: -0.59). In the VAP meta-analysis, we reported a minor asymmetry on publication bias (LFK index: 1.94). Otherwise, there was a major asymmetry in risk of publication bias in cohort meta-analysis about mortality (LFK index: -4.56), ventilator-associated pneumonia (LFK index: -2.10), duration in mechanical ventilation (LFK index: -4.51), ICU length of stay (LFK index: -2.82), and Hospital length of stay (LFK index: -3.83) (Supplementary Material).

### Homogeneity and sensitivity

To address heterogeneity in observational studies, we separated prospective from retrospective cohorts and excluded the only study^22^ that used prolonged intubation as the control group. This analysis showed an increase in mortality in the ET group in prospective cohorts, although this result was based on a major asymmetry in publication bias analysis (RR = 1.74 [1.25, 2.41], I^2^ = 23%, *Q* = 3.87, p = 0.28, LFK index = -4.65). The retrospective cohorts presented no difference between groups and no asymmetry in publication bias (RR = 1.21 [0.50, 2.93], I^2^ = 28%, *Q* = 2.77, p = 0.25, LFK index = -0.20). The benefit of ET in reducing the risk of ventilator-associated pneumonia was sustained when we removed the Siddiqui et al. (2015)^22^ study, and the data were presented with minor asymmetry (LFK index = -1.81) (Supplementary Material).

### GRADE

The force of evidence was considered moderate in cohort studies on ICU length of stay, hospital length of stay, and duration of mechanical ventilation. However, mortality and ventilator-associated pneumonia were low or very low (Table 2).

## Discussion

The impact of early tracheostomy does not appear to be significant on mortality in patients on mechanical ventilation due to neurological involvement. Our results showed that both randomized clinical trials and cohort studies did not provide enough data to define the effectiveness of ET in preventing deaths in severe TBI patients, and these results were also the same as those found for other neurological causes, such as stroke,^29^ acute brain injury,^30^ and spinal cord trauma.^31^

Regarding the incidence of Ventilator-Associated Pneumonia (VAP), the literature reports highly discordant results, not only for TBI. For example, in critically ill patients in the ICU, some studies indicate no difference between early and late tracheostomy.^33^, ^34^, ^35^ Other studies showed benefit from performing an ET in neurological patients;^29^^,^^30^^,^^36^ however, in non-neurological patients, they did not maintain that benefit.^37^

There was a difference in the data on pneumonia cases in the Alali et al. (2014)^26^ study. Franca et al. (2020)^16^ used a propensity-matched analysis excluding deaths; as a result, 213/516 cases were reported in the ET group and 281/516 in the LT group. However, two meta-analyses^15^^,^^17^ used a propensity-matched analysis with deaths; therefore, the number of events was 238/571 in the ET group and 301/571 in the LT group. In our meta-analyses, we used a propensity-matched analysis with deaths and found that early tracheostomy reduced the risk of ventilator-associated pneumonia by 22%.

Time on mechanical ventilation is highly relevant in critically ill care. As in VAP, neurological patients^29^, ^30^, ^31^^,^^38^ show better outcomes with early tracheostomy than critically ill patients in general^34^ or non-neurological patients.^37^ This umbrella supports the results in neurological patients, with moderate-high certainty, and the evidence from cohorts, mainly due to the magnitude of the effect, with an average reduction of 7.53 days in mechanical ventilation time in the ET group. However, the clinical trials failed to confirm that benefit.

Although some studies fail to demonstrate a benefit of early tracheostomy in critically ill patients in reducing ICU length of stay 33,37, most of the literature indicates that patients undergoing ET spend less time in the ICU.^30^^,^^31^^,^^35^^,^^36^^,^^38^ The Umbrella result followed the same direction as the time in mechanical ventilation. It showed a benefit with moderate certainty of evidence, with a mean reduction of 7.34 days in patients undergoing ET, based on an evaluation of 651 patients.

This Umbrella review indicated an average mean reduction of 3.53 days in hospital length of stay in the ET group, with moderate certainty of evidence evaluating 1939 patients. Bertini et al. (2023)^38^ also reported this benefit, but with a smaller magnitude, reducing the average by 1.26 days with PT. As in VAP, ET seems to be better indicated in neurological patients^29^, ^30^, ^31^^,^^38^ than in non-neurological patients.^37^

Outcomes as complications (pneumonia, septicemia, candidemia, pressure ulcers, thromboembolic events, and time on antibiotics), and quality of life (scores regarding neurological function) lacked sufficient data to assess the effectiveness of early tracheostomy, and this should be taken into consideration for future trials.

Our results were limited by lower concordance among reviewers in the risk of bias analysis, although divergences were resolved in consensus meetings, and the selection showed satisfactory concordance among reviewers. Another limitation was the risk of bias analysis in the primary studies, which we did not perform, but the four meta-analyses included did not report major issues in their analyses.

## Conclusions

The evidence included in this umbrella review suggests that early tracheostomy is associated with reduced ICU and Hospital length of stay, time on mechanical ventilation, and ventilator-associated pneumonia. These studies cannot determine the effectiveness of early tracheostomy on mortality and neurological prognosis.

## Data availability statement

The datasets generated and/or analyzed during the current study are available from the corresponding author upon reasonable request.

## Authors’ contributions

Raul Ribeiro de Andrade: Conceptualization (Lead), Data curation (Lead), Formal analysis (Lead), Investigation (Lead), Methodology (Lead), Project administration (Lead), Writing-original draft (Lead).

Edla Vitória Santos Pereira: Data curation (Equal), Formal analysis (Equal).

Igor Hudson Albuquerque e Aguiar: Investigation (Equal).

Olavo Barbosa de Oliveira Neto: Data curation (Supporting), Formal analysis (Supporting), Writing-review & editing (Supporting).

João Gustavo Rocha Peixoto dos Santos: Visualization (Supporting), Writing-review & editing (Supporting).

Fabiano Timbó Barbosa: Conceptualization (Equal), Project administration (Equal), Supervision (Equal), Writing-review & editing (Equal).

Célio Fernando de Sousa-Rodrigues: Formal analysis (Supporting), Investigation (Supporting), Methodology (Supporting), Project administration (Supporting), Supervision (Lead), Writing-review & editing (Supporting).

## Fundings

No funding is involved in this project.

## Conflicts of interest

The authors declare no conflicts of interest.

## Appendix Supplementary materials

Image, application 1

Image, application 2

## Footnotes

Supplementary material associated with this article can be found in the online version at doi:10.1016/j.bjane.2026.844727.
