# Supplementary material for: Early tracheostomy in severe traumatic brain injury: an umbrella systematic review
Source: Braz J Anesthesiol. 2026 Feb 12;76(3):844727. doi: 10.1016/j.bjane.2026.844727 (PMC13010461; doi:10.1016/j.bjane.2026.844727)

**SUPPLEMENTARY MATERIAL**

**SEARCH STRATEGY**

| **PUBMED** | **((((((((((((((((((((((Craniocerebral Trauma) OR (Craniocerebral Traumas)) OR (Trauma, Craniocerebral)) OR (Traumas, Craniocerebral)) OR (Head Injuries)) OR (Injuries, Craniocerebral)) OR (Trauma, Head)) OR (Craniocerebral Injuries)) OR (Craniocerebral Injury)) OR (Injury, Craniocerebral)) OR (Head Trauma)) OR (Head Traumas)) OR (Crushing Skull Injury)) OR (Crushing Skull Injuries)) OR (Brain Injuries, Traumatic)) OR (Traumatic Brain Injuries)) OR (TBI (Traumatic Brain Injury))) OR (Traumatic Encephalopathies)) OR (Traumatic Encephalopathy)) OR (Cerebrovascular Trauma)) OR (Trauma, Cerebrovascular)) OR (Vascular Brain Injury)) AND ((tracheostomy) OR (tracheostomies))** |
| --- | --- |
| **EMBASE** | **('traumatic brain injury'/exp OR 'traumatic brain injury' OR 'head injury') AND 'tracheostomy'** |
| **WEB OF SCIENCE** | **((((((((((((((((((((((ALL=(Craniocerebral Trauma)) OR ALL=(Craniocerebral Traumas)) OR ALL=(Trauma, Craniocerebral)) OR ALL=(Traumas, Craniocerebral)) OR ALL=(Head Injuries)) OR ALL=(Injuries, Craniocerebral)) OR ALL=(Trauma, Head)) OR ALL=(Craniocerebral Injuries)) OR ALL=(Craniocerebral Injury)) OR ALL=(Injury, Craniocerebral)) OR ALL=(Head Trauma)) OR ALL=(Head Traumas)) OR ALL=(Crushing Skull Injury)) OR ALL=(Crushing Skull Injuries)) OR ALL=(Brain Injuries, Traumatic)) OR ALL=(Traumatic Brain Injuries)) OR ALL=(TBI (Traumatic Brain Injury))) OR ALL=(Traumatic Encephalopathies)) OR ALL=(Traumatic Encephalopathy)) OR ALL=(Cerebrovascular Trauma)) OR ALL=(Trauma, Cerebrovascular)) OR ALL=(Vascular Brain Injury)) AND ALL=(tracheostomy)** |
| **SCOPUS** | **ALL ( "Craniocerebral Trauma" ) OR ALL ( "Head Trauma" ) OR ALL ( "Crushing Skull Injury" ) OR ALL ( "Traumatic Brain Injury" ) OR ALL ( "Traumatic Encephalopathy" ) OR ALL ( "Cerebrovascular Trauma" ) AND ALL ( "Tracheostomy" )** |
| **LILACS** | **((Cerebrovascular Trauma) OR (Traumatismos Cerebrovasculares) OR (Traumatismo Cerebrovascular) OR (Lesões Encefálicas Traumáticas) OR (Brain Injuries, Traumatic) OR (Lesiones Traumáticas del Encéfalo) OR (Traumatismos Craniocerebrais) OR (Craniocerebral Trauma) OR (Traumatismos Craneocerebrales)) AND ( (Traqueostomia) OR (Tracheostomy) OR (Traqueostomía))** |
| **CENTRAL**  **COCHRANE** | **"#5 - #1 or #2 or #3 or #4" (Word variations have been searched) #1 MeSH descriptor: [Tracheostomy] explode all trees #2MeSH descriptor: [Tracheotomy] explode all trees #3 Tracheostomies #4 Tracheotomies** |
| **SIGLE** | **Tracheostomy** |
| **CLINICAL TRIALS** | **Traumatic Brain Injury \| Tracheostomy** |

**RISK OF BIAS**

All the included meta-analyses were described very well with low concerns in Domain 3 (ROBIS). At the same time, this meta-analysis did not show concerns in primary studies.

| META-ANALYSIS | DOMAIN | RRA | EVSP | CONSENSUS |
| --- | --- | --- | --- | --- |
| MARRA ET AL. 2021 | DOMAIN 1 | LOW | HIGH | LOW |
|  | DOMAIN 2 | HIGH | HIGH |  |
|  | DOMAIN 3 | LOW | HIGH | LOW |
|  | DOMAIN 4 | HIGH | HIGH |  |
|  | FINAL | HIGH | HIGH |  |
| FRANCA ET AL. 2020 | DOMAIN 1 | LOW | LOW |  |
|  | DOMAIN 2 | HIGH | HIGH |  |
|  | DOMAIN 3 | LOW | LOW |  |
|  | DOMAIN 4 | HIGH | HIGH |  |
|  | FINAL | HIGH | HIGH |  |
| LU ET AL. 2018 | DOMAIN 1 | LOW | HIGH | LOW |
|  | DOMAIN 2 | LOW | LOW |  |
|  | DOMAIN 3 | LOW | LOW |  |
|  | DOMAIN 4 | UNCLEAR | UNCLEAR |  |
|  | FINAL | LOW | HIGH | LOW |
| McCREEDIE ET AL. 2017 | DOMAIN 1 | UNCLEAR | LOW | LOW |
|  | DOMAIN 2 | LOW | LOW |  |
|  | DOMAIN 3 | LOW | LOW |  |
|  | DOMAIN 4 | LOW | LOW |  |
|  | FINAL | LOW | LOW |  |
| AGREEMENT | 70.0% | Kappa: 0.400, IC 95%, 0.116-0.684 | | |

**OVERLAP (CCA)**

We performed a new meta-analysis, including each study just one time to avoid inflating the results.

| **Outcome (CCA)** |  | **Meta-analysis** | | | |
| --- | --- | --- | --- | --- | --- |
|  | **Primary studies** | **McCredie et al. 2017^8^** | **Lu et al. 2018^9^** | **Franca et al. 2020^10^** | **Marra et al. 2021^11^** |
| **Mortality (CCA = 30.9%)** | **Bouderka et al. 2004** |  |  |  |  |
|  | **Dunhan et al. 2014** |  |  |  |  |
|  | **Sugerman et al. 1997** |  |  |  |  |
|  | **Robba et al. 2020** |  |  |  |  |
|  | **Rizk et al. 2011** |  |  |  |  |
|  | **Wang et al. 2012** |  |  |  |  |
|  | **Shibahashi et al. 2017** |  |  |  |  |
|  | **Kahlili et al.2017** |  |  |  |  |
|  | **Alali et al. 2014** |  |  |  |  |
|  | **Ahmed et al. 2007** |  |  |  |  |
|  | **Siddiqui et al.2015** |  |  |  |  |
|  | **Barquist et al. 2006** |  |  |  |  |
|  | **Siddiqui et al.2015** |  |  |  |  |
|  | **Huang et al. 2013** |  |  |  |  |

| **Outcome (CCA)** |  | **Meta-analysis** | | | | | | | | | | |  | | |  |
| --- | --- | --- | --- | --- | --- | --- | --- | --- | --- | --- | --- | --- | --- | --- | --- | --- |
|  | **Primary studies** | **McCredie et al. 2017^8^** | | | **Lu et al. 2018^9^** | | | **Franca et al. 2020^10^** | | | **Marra et al. 2021^11^** | |  | | |  |
| **VAP (CCA = 30.7%)** | **Sugerman et al. 1997** |  | | |  | | |  | | |  | |  | | |  |
|  | **Bouderka et al. 2004** |  | | |  | | |  | | |  | |  | | |  |
|  | **Barquist et al. 2006** |  | | |  | | |  | | |  | |  | | |  |
|  | **Dunhan et al. 2014** |  | | |  | | |  | | |  | |  | | |  |
|  | **Blot et al. 2008** |  | | |  | | |  | | |  | |  | | |  |
|  | **Fayed et al. 2012** |  | | |  | | |  | | |  | |  | | |  |
|  | **Ahmed et al. 2007** |  | | |  | | |  | | |  | |  | | |  |
|  | **Wang et al.2012** |  | | |  | | |  | | |  | |  | | |  |
|  | **Alali et al. 2014** |  | | |  | | |  | | |  | |  | | |  |
|  | **Siddiqui et al.2015** |  | | |  | | |  | | |  | |  | | |  |
|  | **Kahlili et al.2017** |  | | |  | | |  | | |  | |  | | |  |
|  | **Shibahashi et al. 2017** |  | | |  | | |  | | |  | |  | | |  |
|  | **Robba et al. 2020** |  | | |  | | |  | | |  | |  | | |  |
| **Outcome (CCA)** |  | | **Meta-analysis** | | | | | | | | | |  | | |  |
|  | **Primary studies** | | **Lu et al. 2018^9^** | | | **Franca et al. 2020^10^** | | | **Marra et al. 2021^11^** | | | |  | | |  |
| **HOSPITAL (LOS)(CCA = 37.5%)** | **Ahmed et al. 2007** | |  | | |  | | |  | | | |  | | |  |
|  | **Wang et al. 2012** | |  | | |  | | |  | | | |  | | |  |
|  | **Alali et al. 2014** | |  | | |  | | |  | | | |  | | |  |
|  | **Siddiki et al. 2015** | |  | | |  | | |  | | | |  | | |  |
|  | **Kahlili et al. 2017** | |  | | |  | | |  | | | |  | | |  |
|  | **Huang et al. 2013** | |  | | |  | | |  | | | |  | | |  |
|  | **Shibahashi et al. 2017** | |  | | |  | | |  | | | |  | | |  |
|  | **Robba et al. 2020** | |  | | |  | | |  | | | |  | | |  |
| **Outcome (CCA)** |  | | | **Meta-analysis** | | | | | | | | | | | |  |
|  | **Primary studies** | | | **McCredie et al. 2017^8^** | | | **Lu et al. 2018^9^** | | | **Franca et al. 2020^10^** | | **Marra et al. 2021^11^** | | | |  |
| **TIME IN MV (CCA = 18.75%)** | **Sugerman et al. 1997** | | |  | | |  | | |  | |  | | | |  |
|  | **Bouderka et al. 2004** | | |  | | |  | | |  | |  | | | |  |
|  | **Dunhan et al. 2014** | | |  | | |  | | |  | |  | | | |  |
|  | **Blot et al. 2008** | | |  | | |  | | |  | |  | | | |  |
|  | **Terragni et al. 2010** | | |  | | |  | | |  | |  | | | |  |
|  | **Fayed et al. 2012** | | |  | | |  | | |  | |  | | | |  |
|  | **Bösel et al. 2013** | | |  | | |  | | |  | |  | | | |  |
|  | **Barquist et al. 2006** | | |  | | |  | | |  | |  | | | |  |
|  | **Youngi et al. 2021** | | |  | | |  | | |  | |  | | | |  |
|  | **Ahmed et al. 2007** | | |  | | |  | | |  | |  | | | |  |
|  | **Wang et al. 2012** | | |  | | |  | | |  | |  | | | |  |
|  | **Alali et al.2014** | | |  | | |  | | |  | |  | | | |  |
|  | **Siddiki et al. 2015** | | |  | | |  | | |  | |  | | | |  |
|  | **Kahlili et al.2017** | | |  | | |  | | |  | |  | | | |  |
|  | **Shibahashi et al. 2017** | | |  | | |  | | |  | |  | | | |  |
|  | **Robba et al. 2020** | | |  | | |  | | |  | |  | | | |  |
|  |  |  |  |  |  |  |  |  |  |  |  | |  |  |  | |

**MORTALITY**

**Table 1: Meta-analysis results**

| Study | Selected primary studies | Number of participants  (Early vs Late Tracheostomy) | Results |
| --- | --- | --- | --- |
| McCredie 2017 | **RCT**  Sugerman 1997  Bouderka 2004  Barquist 2006  Dunhan 2014 | 140 (Data without details about the group) | RR 1.2 [0.44, 3.30]  I^2^: 37% |
| Lu 2018 | **RCT**  Sugerman 1997  Bouderka 2004  Dunhan 2014  **Observational**  Ahmed 2007  Wang 2012  Alali 2014  Shamin 2015  Kahlili 2017 | RCT:  17/81 vs 8/72  Cohort:  71/716 vs 75/799 | RCT:  OR 2.58 [0.96, 6.96]  I^2^: 0%  Cohort:  OR 1.15 [0.81, 1.63]  I^2^: 0% |
| Franca 2020 | **RCT**  Dunhan 2014  **Observational**  Ahmed 2007  Alali 2014  Kahlili 2017  Shibahashi 2017 | RCT and Cohort  302/2294 vs 177/2312 | RCT and Cohort:  Risk Difference 0.03 [-0.02, 0.07]  I^2^: 69% |
| Marra 2021 | **RCT**  Sugerman 1997  Dunhan 2014  **Observational**  Ahmed 2007  Rizk 2011  Huang 2013  Alali 2014  Kahlili 2017  Shibahashi 2017  Robba 2020 | RCT  5/50 vs 1/41  Cohort:  319/2509 vs 189/2597 | RCT  OR 3.154 [0.456, 21.695]  I^2^: 0%  Cohort  OR 1.505 [0.993, 2.279]  I^2^: 49.435% |

**Table 2: RCT and Cohort results**

| **RCT** | | | |
| --- | --- | --- | --- |
| **STUDY** | ET GROUP | LT/PI GROUP | DAY OF ET |
| **BOUDERKA 2004** | 12/31 | 7/31 | 5-6 DAYS |
| **DUNHAN 2014** | 0/15 | 0/9 | 3-5 DAYS |
| **SURGERMAN 1997** | 5/35 | 1/32 | 3-5 DAYS |
| **RETROSPECTIVE COHORTS** | | | |
| **STUDY** | ET GROUP | LT/PI GROUP | DAY OF ET |
| **AHMED & KU 2007** | 4/27 | 1/28 | ≤ 7 DAYS |
| **ALALI 2014** | 48/571 | 39/571 | ≤ 8 DAYS |
| **SHIBAHASHI 2017** | 1/40 | 4/51 | ≤ 72 HOURS |
| **PROSPECTIVE COHORTS** | | | |
| **STUDY** | ET GROUP | LT/PI GROUP | DAY OF ET |
| **RIZK 2011** | 238/1577 | 111/1527 | ≤ 7 DAYS |
| **WANG 2012** | 2/16 | 4/50 | ≤ 7 DAYS |
| **SIDDIQUI 2015** | 4/49 | 9/51 | ≤ 7 DAYS |
| **KHALILI 2017** | 10/53 | 18/99 | ≤ 6 DAYS |
| **ROBBA 2020** | 12/180 | 11/253 | ≤ 7 DAYS |
| ET: Early tracheostomy.  LT: Late tracheostomy.  PI: Prolonged intubation. | | | |

**VENTILATOR-ASSOCIATED PNEUMONIA**

**Table 3: Meta-analysis results**

| Study | Selected primary studies | Number of participants  (Early vs Late Tracheostomy) | Results |
| --- | --- | --- | --- |
| McCredie 2017 | **RCT**  Sugerman 1997  Bouderka 2004  Barquist 2006  Dunhan 2014  Blot 2008  Fayed 2012 | 275 (Data without details about the group) | RR 0.89 [0.65, 1.21]  I^2^: 54% |
| Lu 2018 | **RCT**  Sugerman 1997  Bouderka 2004  Dunhan 2014  **Observational**  Ahmed 2007  Wang 2012  Alali 2014  Shamin 2015  Kahlili 2017 | RCT:  42/81 vs 40/72  Cohort:  306/716 vs 444/799 | RCT:  OR 0.89 [0.47, 1.68]  I^2^: 0%  Cohort:  OR 0.62 [0.51, 0.77]  I^2^: 0% |
| Franca 2020 | **RCT**  Dunhan 2014  **Observational**  Ahmed 2007  Alali 2014  Kahlili 2017  Shibahashi 2017 | RCT and Cohort:  272/651 vs 379/703 | RCT and Cohort:  RR 0.78 [0.70, 0.88]  I^2^: 0% |
| Marra 2021 | **Observational**  Ahmed 2007  Wang 2012  Alali 2014  Kahlili 2017  Shibahashi 2017  Robba 2020 | Cohort  346/887 vs 533/1052 | Cohort  OR 0.623 [0.518, 0.750]  I^2^: 0% |

**Table 4: RCT and Cohort results**

| **RCT** | | | |
| --- | --- | --- | --- |
| **STUDY** | ET GROUP | LT/PI GROUP | DAY OF ET |
| **BOUDERKA 2004** | 8/31 | 19/31 | 5-6 DAYS |
| **DUNHAN 2014** | 7/15 | 4/9 | 3-5 DAYS |
| **SURGERMAN 1997** | 17/35 | 17/32 | 3-5 DAYS |
| **RETROSPECTIVE COHORTS** | | | |
| **STUDY** | ET GROUP | LT/PI GROUP | DAY OF ET |
| **AHMED & KU 2007** | 11/27 | 14/28 | ≤ 7 DAYS |
| **ALALI 2014** | 238/571 | 301/571 | ≤ 8 DAYS |
| **SHIBAHASHI 2017** | 13/40 | 21/51 | ≤ 72 HOURS |
| **PROSPECTIVE COHORTS** | | | |
| **STUDY** | ET GROUP | LT/PI GROUP | DAY OF ET |
| **WANG 2012** | 7/16 | 38/50 | ≤ 7 DAYS |
| **SIDDIQUI 2015** | 22/49 | 32/51 | ≤ 7 DAYS |
| **KHALILI 2017** | 28/53 | 59/99 | ≤ 6 DAYS |
| **ROBBA 2020** | 49/180 | 100/253 | ≤ 7 DAYS |
| ET: Early tracheostomy.  LT: Late tracheostomy.  PI: Prolonged intubation. | | | |

**DURATION OF MECHANICAL VENTILATION**

**Table 5: Meta-analysis results**

| Study | Selected primary studies | Number of participants  (Early vs Late Tracheostomy) | Results |
| --- | --- | --- | --- |
| McCredie 2017 | **RCT**  Sugerman 1997  Bouderka 2004  Barquist 2006  Blot 2008  Terragni 2010  Fayed 2012  Bösel 2013  Dunhan 2014  Youngi 2021 | 412 (Data without details about the group) | MD -2.72 [-4.15, -2.19]  I^2^: 0% |
| Lu 2018 | **RCT**  Bouderka 2004  Dunhan 2014  **Observational**  Ahmed 2007  Wang 2012  Alali 2014  Siddiki 2015  Kahlili 2017 | RCT and Cohort:  709 vs 740 | RCT and Cohort:  Mean Difference  -4.92 [-6.82, -3.02]  I^2^: 51% |
| Franca 2020 | **RCT**  Dunhan 2014  **Observational**  Ahmed 2007  Alali 2014  Shibahashi 2017 | RCT and Cohort:  598 vs 604 | RCT and Cohort:  Mean Difference  -4.15 [-6.30, -1.99]  I^2^: 85% |
| Marra 2021 | **RCT**  Dunhan 2014  **Observational**  Ahmed 2007  Alali 2014  Shibahashi 2017  Robba 2020 | RCT and Cohort  NA | RCT and Cohort:  Mean Difference  -4.866 [-6.981, -2.751]  I^2^: 93.203% |

**Table 6: RCT and Cohort results**

| **RCT** | | | | |
| --- | --- | --- | --- | --- |
| **STUDY** | ET GROUP | LT/PI GROUP | DAY OF ET |  |
| **BOUDERKA 2004** | 14.5 ± 7.3d / 31p | 17.5 ± 10.6d / 31p | 5-6 DAYS |  |
| **DUNHEM 2014** | 14.1 ± 5.7d / 15p | 19 ± 11.3d / 32 p | 3-5 DAYS |  |
| **RETROSPECTIVE COHORTS** | | | |  |
| **STUDY** | ET GROUP | LT/PI GROUP | DAY OF ET |  |
| **AHMED & KU 2007** | 15.7 ± 6d / 27 p | 25.8 ± 11.8 d / 28p | ≤ 7 DAYS |  |
| **ALALI 2014** | 21.4 ± 10.45d/571p* | 24.9 ± 5.95d/ 571p* | ≤ 8 DAYS |  |
| **SHIBAHASHI 2017** | 5 ± 1.54d / 40p* | 8 ± 3.05d/ 51p* | ≤ 72 HOURS |  |
| **PROSPECTIVE COHORTS** | | | |  |
| **STUDY** | ET GROUP | LT/PI GROUP | DAY OF ET |  |
| **WANG 2012** | 13.7 ± 7.3d / 16p | 23.4 ± 11d / 50p | ≤ 7 DAYS |  |
| **ROBBA 2020** | 12.35 ± 6.73d/180p* | 19.63 ± 10.29d/253p* | ≤ 7 DAYS |  |
| ET: Early tracheostomy.  LT: Late tracheostomy.  PI: Prolonged intubation.  Data in Mean ± Standard Deviation / participants.  * Data converted of Median and Interquartil interval from primary studies by Wan et al. 2014. | | | |  |
|  | | | |  |

**ICU LENGHT OF STAY**

**Table 7: Meta-analysis results**

| **Study** | **Selected primary studies** | **Number of participants**  **(Early vs Late Tracheostomy)** | **Results** |
| --- | --- | --- | --- |
| **McCredie et al. 2017** | **RCT**  Sugerman 1997  Blot 2008  Terragni 2010  Bösel 2013  Youngi 2021 | 326 (Data without details about the group) | MD -2.55 [-4.59, -0.50]  I^2^: 0% |
| **Lu et al. 2018** | **RCT**  Sugerman 1997  Bouderka 2004  Dunhan 2014  **Cohorts**  Ahmed 2007  Wang 2012  Siddiki 2015  Kahlili 2017 | RCT and Cohorts:  226 vs 300 | RCT and Cohorts:  Mean Difference  -3.08 [-3.75, -2.41]  I^2^: 38% |
| **Franca 2020** | **Cohorts**  Ahmed 2007  Huang 2013  Alali 2014  Kahlili 2017  Shibahashi 2017 | Cohorts:  647 vs 721 | Cohorts:  Mean Difference  -5.87 [-8.74, -3.00]  I^2^: 83% |
| **Marra 2021** | **Cohorts**  Ahmed 2007  Huang 2013  Alali 2014  Shibahashi 2017  Kahlili 2017  Robba 2020 | Cohorts  (Data without details about the group) | Cohorts:  Mean Difference  -5.96 [-7.99, -3.92]  I^2^: 88.661% |

**Table 8: RCT and Cohort results**

| **RCT** | | | | |
| --- | --- | --- | --- | --- |
| **STUDY** | ET GROUP | LT/PI GROUP | DAY OF ET |  |
| **SURGERMAN 1997** | 16 ± 5.9 d / 35 p | 19 ± 11.3 d / 32 p | 3-5 DAYS |  |
| **RETROSPECTIVE COHORTS** | | | |  |
| **STUDY** | ET GROUP | LT/PI GROUP | DAY OF ET |  |
| **AHMED & KU 2007** | 19 ± 7.7d / 27p | 25.8 ± 11.8 d / 28p | ≤ 7 DAYS |  |
| **ALALI 2014** | 13.7 ± 5.95d / 571p* | 19.7 ± 7.43 / 571p* | ≤ 8 DAYS |  |
| **SHIBAHASHI 2017** | 10 ± 4.61d / 40p* | 12.06 ± 3.81d / 51p* | ≤ 72 HOURS |  |
| **PROSPECTIVE COHORTS** | | | |  |
| **STUDY** | ET GROUP | LT/PI GROUP | DAY OF ET |  |
| **WANG 2012** | 14.9 ± 8.9d / 16p | 22.1 ± 7.6d / 50p | ≤ 7 DAYS |  |
| **KHALILI 2017** | 26.79 ± 13.16d/ 53p | 34.92 ± 20.07d/ 99p | ≤ 6 DAYS |  |
| **ROBBA 2020** | 19.6 ± 19.9d / 180p | 26.7 ± 12.5d / 253p | ≤ 7 DAYS |  |
| ET: Early tracheostomy.  LT: Late tracheostomy.  PI: Prolonged intubation.  Data in Mean ± Standard Deviation / participants.  * Data converted of Median and Interquartil interval from primary studies by Wan et al. 2014. | | | |  |
|  | | | |  |

**HOSPITAL LENGTH OF STAY**

**Table 9: Meta-analysis results**

| **Study** | **Selected primary studies** | **Number of participants**  **(Early vs Late Tracheostomy)** | **Results** |
| --- | --- | --- | --- |
| **Lu et al. 2018** | **Cohorts**  Ahmed 2007  Wang 2012  Alali 2014  Siddiki 2015  Kahlili 2017 | Cohorts:  716 vs 798 | Cohorts:  Mean Difference  -4.79 [-8.63, -0.94]  I^2^: 59% |
| **Franca 2020** | **Cohorts**  Huang 2013  Alali 2014  Kahlili 2017  Shibahashi 2017 | Cohorts:  620 vs 692 | Cohorts:  Mean Difference  -6.68 [-8.03, -5.32]  I^2^: 0% |
| **Marra 2021** | **Cohorts**  Ahmed 2007  Alali 2014  Shibahashi 2017  Kahlili 2017  Robba 2020 | Cohorts  (Data without details about the group) | Cohorts:  Mean Difference  -6.97 [-8.25, -5.68]  I^2^: 0% |

**Table 10: RCT and Cohort results**

| **RETROSPECTIVE COHORTS** | | | |
| --- | --- | --- | --- |
| **STUDY** | ET GROUP | LT/PI GROUP | DAY OF ET |
| **AHMED & KU 2007** | 24.36 ± 5.48d / 27p* | 28 ± 6.25d / 28p* | ≤ 7 DAYS |
| **ALALI 2014** | 21.4 ± 10.41d/571p* | 24.9 ± 5.95/571p* | ≤ 8 DAYS |
| **SHIBAHASHI 2017** | 52.64 ± 19.22d/40p* | 56.29 ± 16.78d/51p | ≤ 72 HOURS |
| **PROSPECTIVE COHORTS** | | | |
| **STUDY** | ET GROUP | LT/PI GROUP | DAY OF ET |
| **WANG 2012** | 38.0 ± 21.4d/ 16p | 46.8 ± 22d / 50p | ≤ 7 DAYS |
| **ROBBA 2020** | 35.1 ± 34.4d /180p | 34.7 ± 33.6 d / 253 p | ≤ 7 DAYS |
| **KHALILI 2017** | 38.58 ± 20.18d/ 53p | 46.40 ± 24.56d/ 99p | ≤ 6 DAYS |
| ET: Early tracheostomy.  LT: Late tracheostomy.  PI: Prolonged intubation.  Data in Mean ± Standard Deviation / participants.  * Data converted of Median and Interquartil interval from primary studies by Wan et al. 2014. | | | |
|  | | | |

**RISK OF PUBLICATION BIAS**

**In RCTs Meta-analysis**

- **MORTALITY**


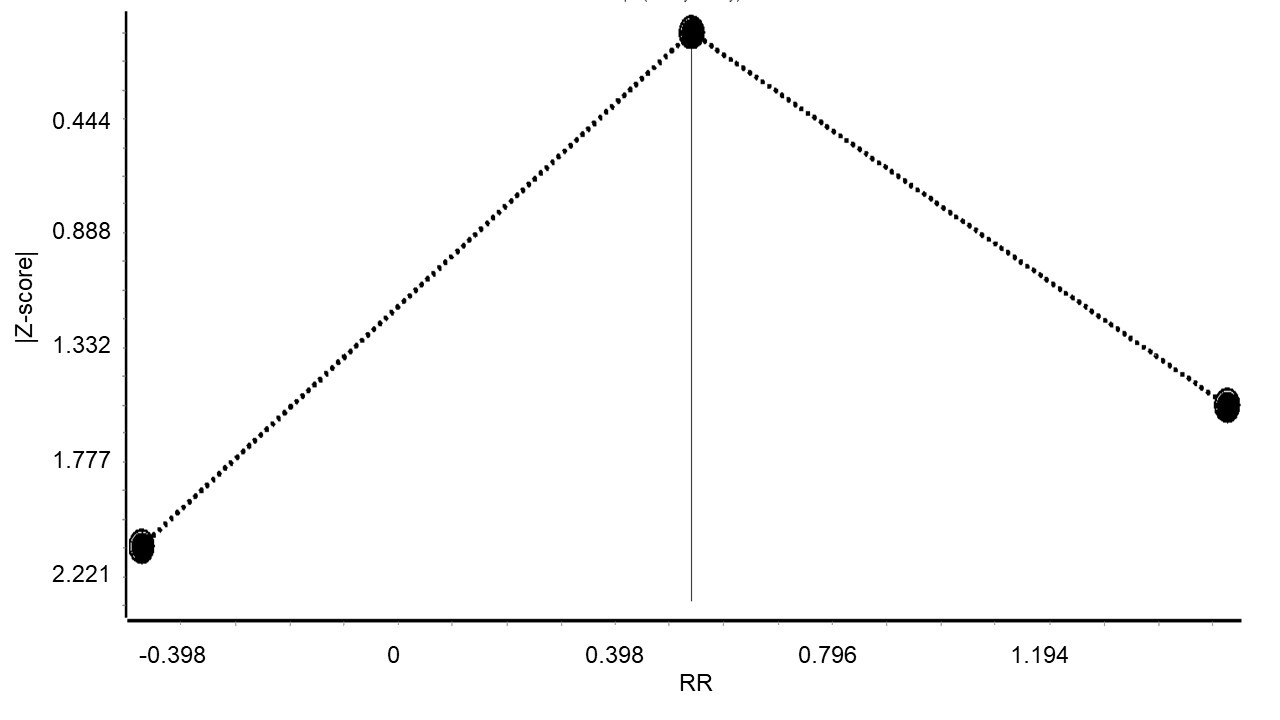


- **VENTILATOR-RELATED PNEUMONIA**


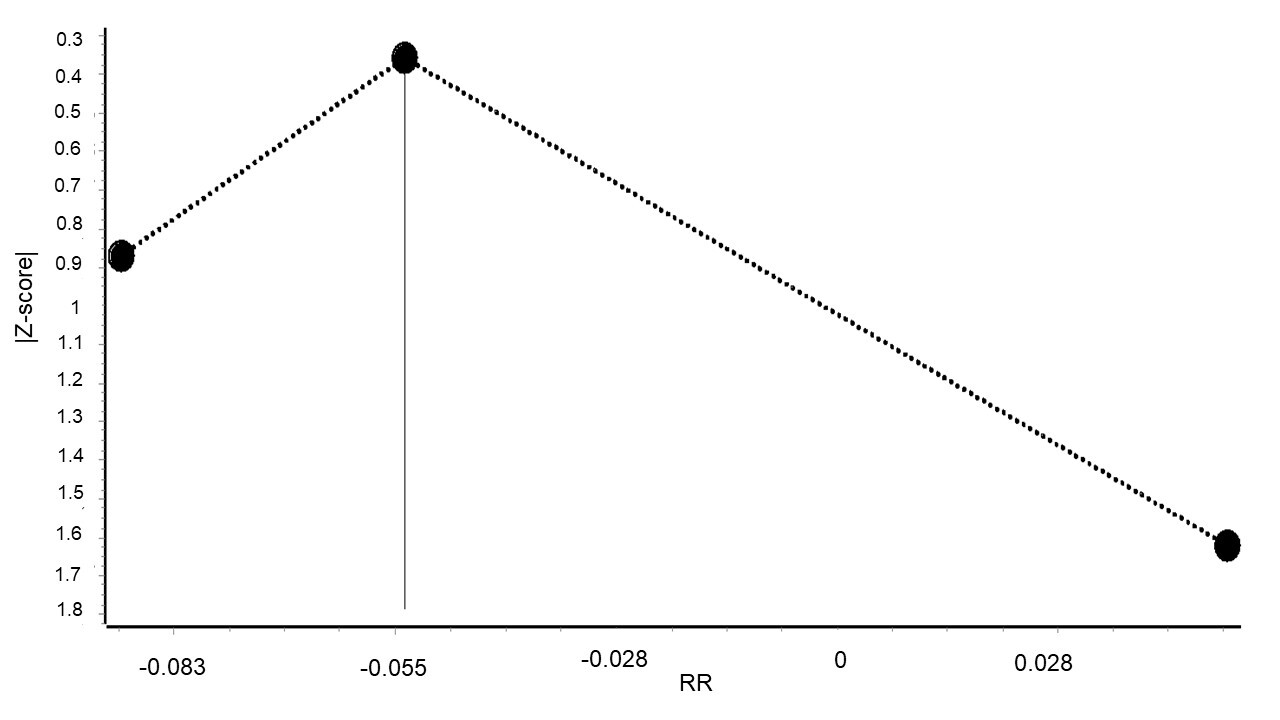


**In Cohorts meta-analysis**

- **MORTALITY**


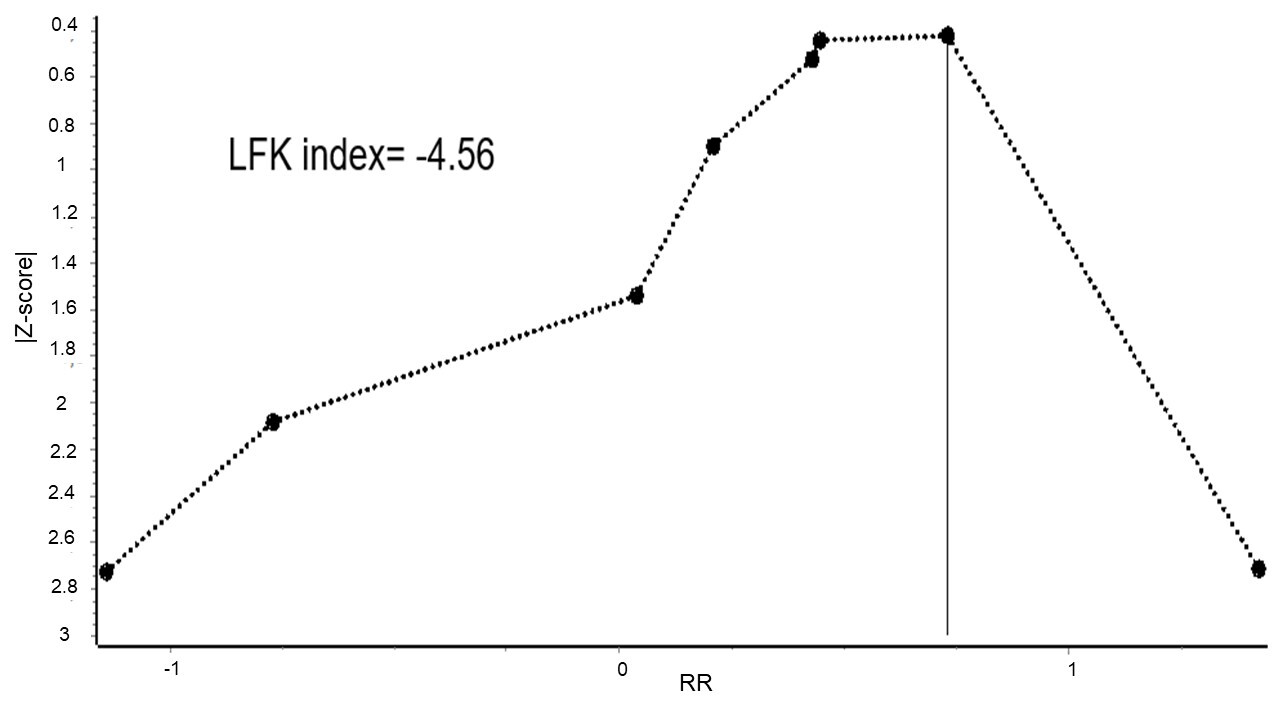


- **VENTILATOR-RELATED PNEUMONIA**


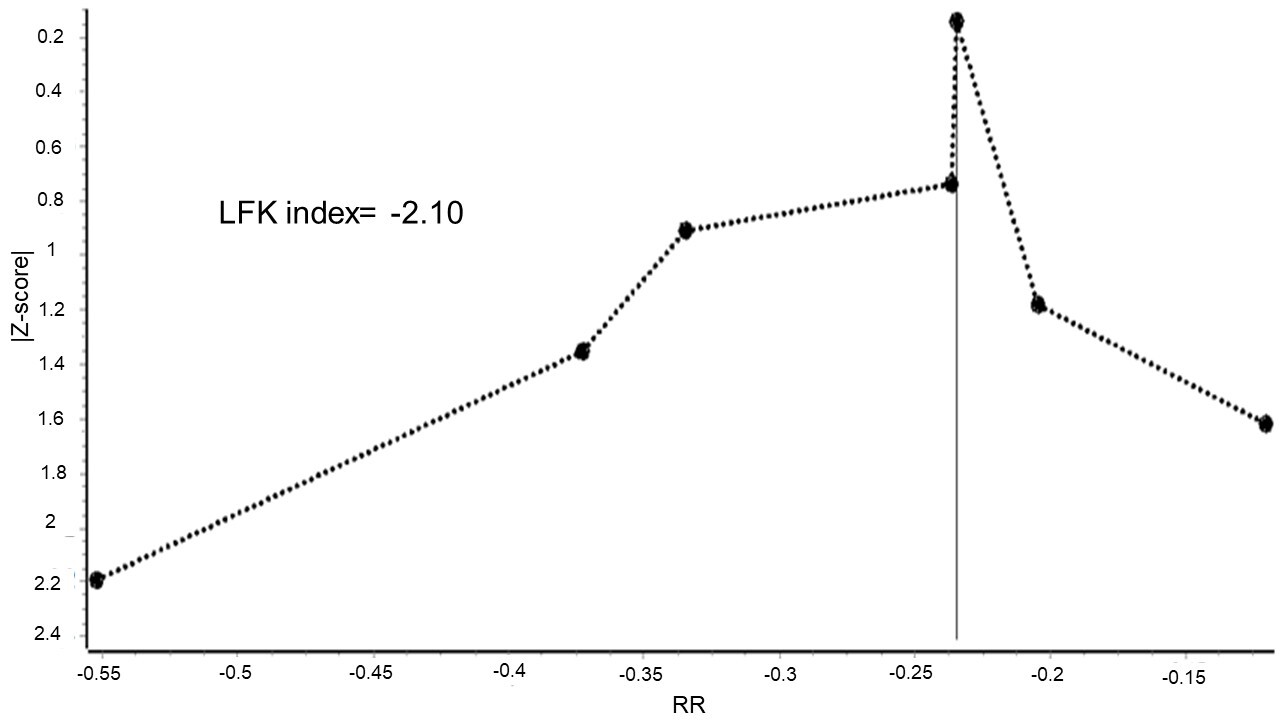


- **DURATION IN MECHANICAL VENTILATION**


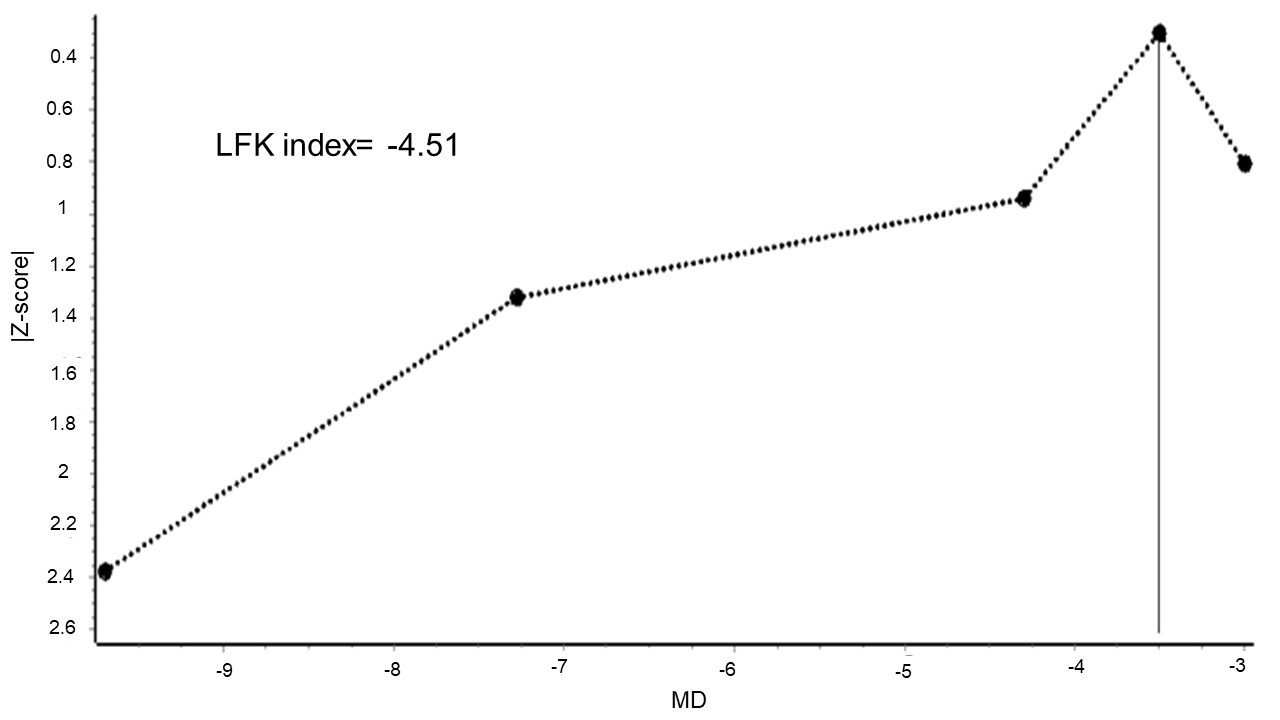


- **ICU LENGHT OF STAY**


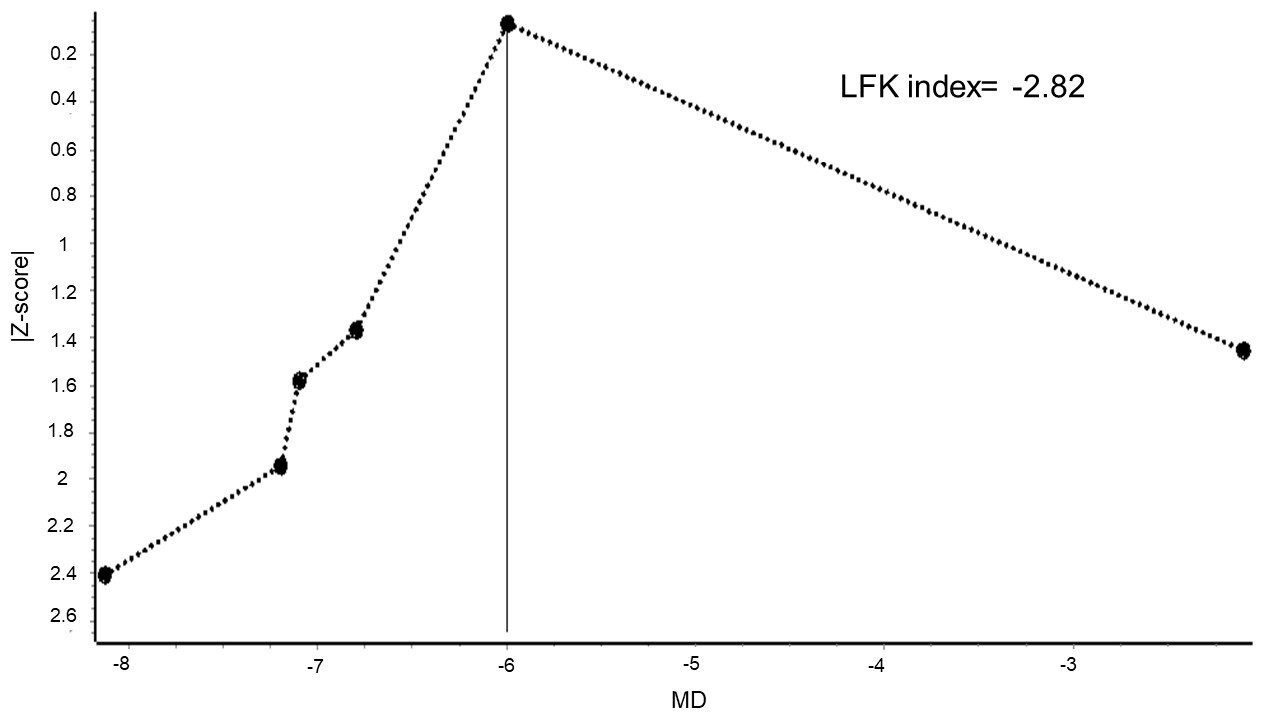


- **HOSPITAL LENGHT OF STAY**


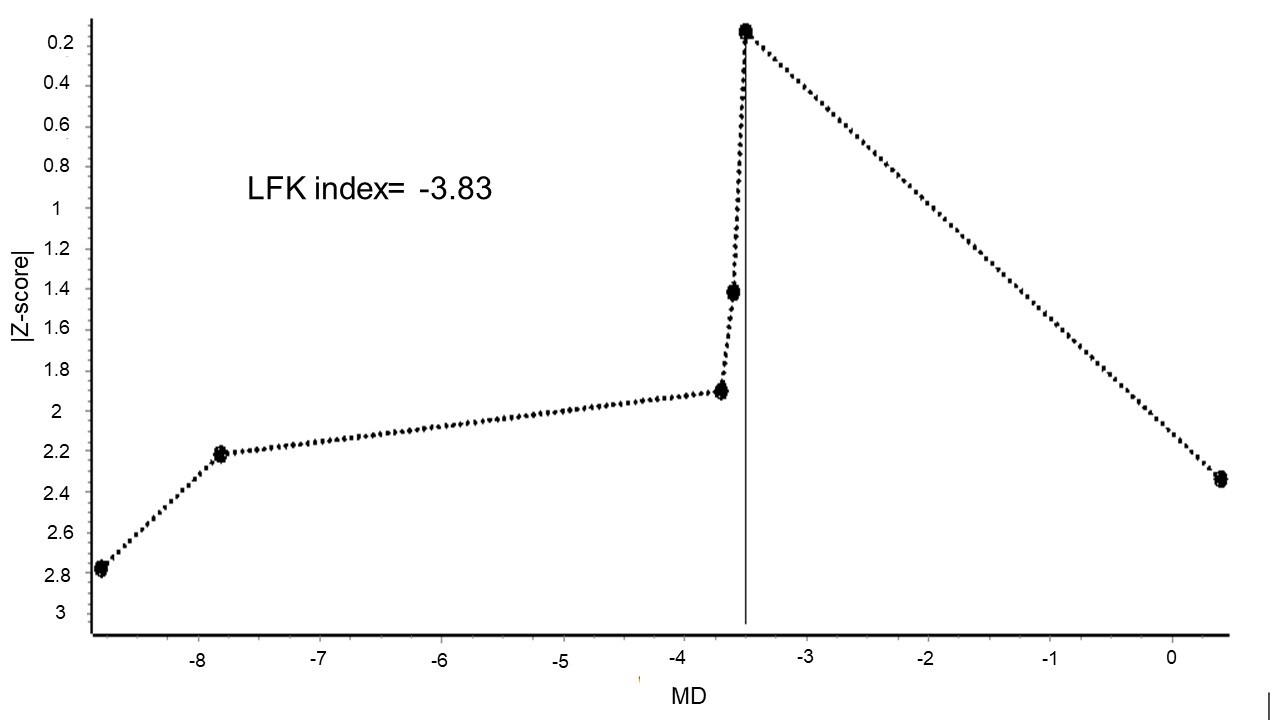

Supplement: Supplementary file 1 [file mmc1.docx]
