# Supplementary material for: Early tracheostomy in severe traumatic brain injury: an umbrella systematic review
Source: Braz J Anesthesiol. 2026 Feb 12;76(3):844727. doi: 10.1016/j.bjane.2026.844727 (PMC13010461; doi:10.1016/j.bjane.2026.844727)
Supplement: Supplementary file 2 [file mmc2.pdf]

International Platform of Registered Systematic Review and Meta-analysis Protocols

INPLASY PROTOCOL

To cite: Andrade et al. Effectiveness of Early Tracheostomy compared with Late Tracheostomy Or Prolonged Orotracheal Intubation in Traumatic Brain Injury: Protocol of Umbrella Review. Inplasy protocol 202280096. doi: 10.37766/inplasy2022.8.0096

Received: 24 August 2022

Published: 24 August 2022

Corresponding author:
Raul Ribeiro de Andrade

raulrandrade.med@gmail.com

Author Affiliation:
Universidade Federal de Alagoas.

Support: **The authors.**

Review Stage at time of this submission: **Formal screening of search results against eligibility criteria.**

Conflicts of interest:
None declared.

Effectiveness of Early Tracheostomy compared with Late Tracheostomy Or Prolonged Orotracheal Intubation in Traumatic Brain Injury: Protocol of Umbrella Review

Andrade, RR[1]; Pereira, EVS[2]; Aguiar, IHA[3]; Oliveira Neto, OB[4]; Barbosa, FT; Santos, JGRP; Sousa-Rodrigues, CF.

Review question / Objective: **What is the effectiveness of Early Tracheostomy compared with Late Tracheostomy Or Prolonged Orotracheal Intubation in Traumatic Brain Injury?**
Eligibility criteria: **The inclusion criteria are (P) studies with patients above 18 years old, male or female, who had a severe traumatic brain injury and who need advanced airway support; (I) patient undergoing early tracheostomy (less than 10 days of orotraqueal intubation); (C) patient undergoing late tracheostomy (after 10 days of orotraqueal intubation) or undergoing prolonged intubation; (O) With data about mortality, time on ICU stay, on Hospital stay and time free of mechanical ventilation, complications related a health care services (pneumonia, septicemia, candidemia, Pressure ulcers, thromboembolic events and time using antibiotics), Quality of life (scores about neurological functions); e (S) Systematic reviews. No language restrictions. The exclusion criteria are data about mortality without data about time and follow up (In Hospital or after discharge?). We will contact the authors of studies without data enough to make a decision or without full text available, If we do not have answers we will exclude the study.**

INPLASY registration number: This protocol was registered with the International Platform of Registered Systematic Review and Meta-Analysis Protocols (INPLASY) on 24 August 2022 and was last updated on 24 August 2022 (registration number INPLASY202280096).

INTRODUCTION

Review question / Objective: **What is the effectiveness of Early Tracheostomy compared with Late Tracheostomy Or Prolonged Orotracheal Intubation in Traumatic Brain Injury?**

Condition being studied: **Traumatic Brain Injury (TBI) is every traumatic anatomical ou functional injury that affects brain, skull**

Andrade et al. Inplasy protocol 202280096. doi:10.37766/inplasy2022.8.0096 Downloaded from https://inplasy.com/inplasy-2022-8-0096/

and/or vessels related to them. TBI is a public health problem that involves over 50 million people per year in Worldwide.

METHODS

Participant or population: Traumatic Brain Patients with advanced airway support.

Intervention: Early tracheostomy (less than 10 days of orotracheal intubation)

Comparator: Late tracheostomy (more than 10 days of orotracheal intubation) OR Prolonged Orotracheal Intubation.

Study designs to be included: Systematic review with metanalyses.

Eligibility criteria: The inclusion criteria are (P) studies with patients above 18 years old, male or female, who had a severe traumatic brain injury and who need advanced airway support; (I) patient undergoing early tracheostomy (less than 10 days of orotraqueal intubation); (C) patient undergoing late tracheostomy (after 10 days of orotraqueal intubation) or undergoing prolonged intubation; (O) With data about mortality, time on ICU stay, on Hospital stay and time free of mechanical ventilation, complications related a health care services (pneumonia, septicemia, candidemia, Pressure ulcers, thromboembolic events and time using antibiotics), Quality of life (scores about neurological functions); e (S) Systematic reviews. No language restrictions. The exclusion criteria are data about mortality without data about time and follow up (In Hospital or after discharge?). We will contact the authors of studies without data enough to make a decision or without full text available, If we do not have answers we will exclude the study.

Information sources: PUBLISHED DATABASES (Medline by PUBMED, Lilacs, Dare-Cochrane, Scopus by Elsevier, Web Of Science e Embase by Elsevier) NON-PUBLISHED (Open Grey by Sigle; Clinical Trial Register at the International Clinical Trials Registry Platform) (Referencies of the selected studies).

Main outcome(s): Mortality: Number of deaths on Hospital or after discharge related with traumatic brain injury.

Additional outcome(s): Secondaries outcomes: * frequency of complications related to UCI stay and duration of mechanical ventilation (number of events of Pneumonia, Septicemia, Candidemia, Pressure ulcers, thromboembolic events) * time (in days) on ICU stay * time (in days) on Hospital stay * time (in days) free of mechanical ventilation * time (in days) of using antibiotics * Quality of life (scores about neurological functions) Tertiary variables (Age, sex, time of follow up, neurosurgical interventions, APACHE II, Glasgow Coma Scale, Injury Severity Score).

Data management: One reviewer (RRA) will select the studies and other reviewer (IHAA) will check the selection. These reviewers will solve disagreements by means of meetings and consensus. A third and more experienced reviewer (OBON) will be consulted when disagreements persist. The same two reviewers (RRA and OBON) will also perform data extraction using pre established forms, where the following data will be extracted: - Title
- Authors
- Journal
- Number
- Year
- Pages
- Database found
*Specific data:
- Aim
- Type of study
- Inclusion and exclusion criteria
- Patients' features
- Intervention and comparison
- Description of outcomes
- Assessment of outcomes
- Risk of bias of the systematic review using the Robis Tool
- Methods of study selection
- Methods of methodological quality assessment or risk of bias assessment of primary studies.
* Statistical data:
- Continuous outcomes

Andrade et al. Inplasy protocol 202280096. doi:10.37766/inplasy2022.8.0096 Downloaded from https://inplasy.com/inplasy-2022-8-0096/

- Categorical outcomes
- Results from meta-analysis
* Conclusions of the study- Title
- Authors
- Journal
- Number
- Year
- Pages
- Database found
*Specific data:
- Aim
- Type of study
- Inclusion and exclusion criteria
- Patients' features
- Intervention and comparison
- Description of outcomes
- Assessment of outcomes
- Methods of study selection
- Risk of bias analyses
* Statistical data:
- Continuous outcomes
- Categorical outcomes
* Conclusions of the study

Quality assessment / Risk of bias analysis: According to ROBIS tool, the risk of bias can be classified as high, low or unclear.

Strategy of data synthesis: The Cohen's kappa statistics will be performed to measure the level of agreement between reviewers for the selection of eligible studies and for the risk of bias assessment. MetaXL 5.3 (Epigear, Queensland, Australia) will be used to metanalyses. To dichotomic outcomes we will calculate Relative Risk (RR) for prospective studies, and Odds Ratio (OR) for retrospective studies (Confidence Interval 95%). To continous outcomes will be calculated Mean Difference or standardized mean difference (Confidence Interval 95%). Predicting a possible heterogeneity between studies, the random effects model will be used. Heterogenity will be analysed by Q's Cochrane with p value related and will be measured by Higgins Test (I2). Will be reported as small (I270%). The publication bias will be accessed with DOI-plot and LFK index. To solve heterogenity will be peformed a sensitivy analysis.

Subgroup analysis: Time of follow up, Age, Glasgow Coma Scale, Injury Severety Score, Type of complications.

Sensitivity analysis: Based on risk of bias/ Quality assessment Published vs Non Published studies.

Language restriction: No restrictions.

Country(ies) involved: Brazil.

Keywords: Early Tracheostomy ; Traumatic Brain Injury ; Mortality.

Dissemination plans: Journal of Neurotrauma, Intensive Care and Revista Brasileira de Medicina Intensiva.

Contributions of each author:

Author 1 - RAUL RIBEIRO DE ANDRADE - MAIN REVIEWER, RESPONSIBLE FOR PROTOCOL, SEARCH, SELECTION, RISK OF BIAS, DATA EXTRACTION, DRAFT OF MANUSCRIPT, DISSEMINATION EXTRACTION, DRAFT OF MANUSCRIPT, DISSEMINATION.
Email: raulrandrade.med@gmail.com
Author 2 - EDLA VITÓRIA SANTOS PEREIRA - SECOND REVIEWER, RESPONSIBLE FOR RISK OF BIAS DATA EXTRACTION.
Email: edla.pereira@foufal.ufal.br
Author 3 - IGOR HUDSON ALBUQUERQUE E AGUIAR - THIRD REVIEWER, RESPONSIBLE FOR SEARCH, SELECTION.
Email: igor.aguiar@foufal.ufal.br
Author 4 - OLAVO BARBOSA DE OLIVEIRA NETO - METODOLOGICAL EXPERT TO SOLVE THE CONFLICTS OF SELECTION AND RISK OF BIAS.
Email: olavobarbosa91@gmail.com
Author 5 - FABIANO TIMBÓ BARBOSA - RESPONSIBLE FOR PROTOCOL AND DISSEMINATION.
Author 6 - JOÃO GUSTAVO ROCHA PEIXOTO DOS SANTOS - REVIEW THE MANUSCRIPT WITH EXPERT APPROACH.
Author 7 - CÉLIO FERNANDO DE SOUSA-RODRIGUES - RESPONSIBLE FOR PROTOCOL AND DRAFT THE MANUSCRIPT.

Andrade et al. Inplasy protocol 202280096. doi:10.37766/inplasy2022.8.0096 Downloaded from https://inplasy.com/inplasy-2022-8-0096/
